# Functional Genome Screening to Elucidate the Colistin Resistance Mechanism

**DOI:** 10.1038/srep23156

**Published:** 2016-03-18

**Authors:** Mohit Kumar, Ashutosh Gupta, Rajesh Kumar Sahoo, Jayanti Jena, Nagen Kumar Debata, Enketeswara Subudhi

**Affiliations:** 1 Biotechnology and Bioinformatics, NIIT University, Neemrana, Rajasthan 301705, India; 2 Department of Microbiology, IMS & SUM Hospital, Siksha ‘O’ Anusandhan University, Kalinga Nagar, Bhubaneswar, Odisha 751003, India; 3 Centre of Biotechnology, Siksha ‘O’ Anusandhan University, Khandagiri, Bhubaneswar, Odisha 751003, India

## Abstract

Antibiogram profile of 1590 clinical bacterial isolates based on thirteen different antimicrobial compounds showed that 1.6% of the bacterial isolates are multidrug resistant. Distribution pattern based on 16S rRNA sequence analysis showed that *Pseudomonas aeruginosa* constituted the largest group (83.6%) followed by *Burkholderia pseudomallei* sp. A191 (5.17%), *Staphylococcus* sp. A261 (3.45%). Among the various antibiotics used, colistin appeared to be the most effective against the Gram negative bacteria. *Burkholderia pseudomallei* sp. A191 and *Pseudomonas aeruginosa* sp. A111 showed resistance to 1500 μg/ml and 750 μg/ml of colistin respectively which constitutes 7.7% of the bacterial population. A functional genomics strategy was employed to discover the molecular support for colistin resistance in *Burkholderia pseudomallei* sp. A191. A pUC plasmid-based genomic expression library was constructed with an estimated library size of 2.1 × 10^7^bp. Five colistin resistant clones were obtained after functional screening of the library. Analysis of DNA sequence of five colistin resistant clones showed homology to two component regularity systems (TCRS) encoding for a histidine kinase (mrgS) and its regulatory component (mrgR). Cross complementation assay showed that mutations in *mrgS* were sufficient enough to confer colistin resistant phenotype in a sensitive strain.

Antimicrobial resistance (AMR) is a global public health crisis and is of great concern of every country irrespective of their socio-economic status^1^. Hospitals, more particularly intensive care units, are major sites of origin for the development and evolution of antibiotic resistant bacteria^2^. In developing countries, hospitals are crowded with debilitated patients who are administered with heavy doses of broad-spectrum antibiotics often without diagnosing specific infecting organism and without following any proper guidelines leading to an ineffective treatment. This brings in a threat to the patient survival and also to curb spread of infection^3^.

Outbreaks of infections with multi drug resistant (MDR) strains in intensive care unit settings have been reported in several countries across the world. The treatment of these infections has become difficult due to growing prevalence of pan drug resistance (PDR). Although, in these resistant strains, colistin (also known as polymyxin E) is often considered as the last resort of treatment, there are few reports on emergence of colistin-resistance in different corners of the globe^4^,^5^.

Colistin disrupts membrane integrity by displacing Mg^2+^ and Ca^2+^ cations from the outer membrane leading to cell lysis^6^. Bacteria showing resistant to colistin may find ways out to modify the lipopolysaccharide (LPS), particularly through displacement of the phosphate groups of lipid A to 4-amino4-deoxy-l-arabinose and/or phosphoethanolamine resulting in reduction in the electrostatic affinity between the cationic colistin and anionic LPS. Mutations in the transcriptional regulatory systems controlling these LPS modifications are a common genetic mechanism probably leading to colistin resistance^7^. Few reports have been surfaced out in Indian subcontinent about the colistin resistance however the mechanism of its resistance has not been figured out so far^8^. Moreover, no report is available on the genetic mechanisms of colistin resistance in *Burkholderia* species. In the present study, we investigated the mechanism of colistin resistance by functional genome screening in *Burkholderia pseudomallei*.

## Results

A total of 1590 bacterial isolates were collected from clinical samples (wound, burn injuries, sputum, pus, urine and head injuries) isolated from patients admitted to various wards of the hospital. Average age of the hospitalized patients was found to be 34 years of which 42.3% of the patients were female and 57.7% were male (Table 1).

**Figure 1:**
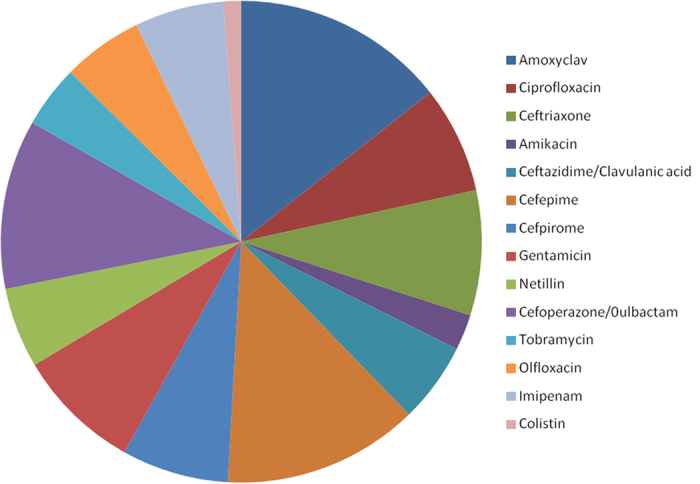
Antibiogram of the selected isolates.

Clinical isolates which showed resistance to more than any three antibiotics (1.6%) were selected for further study. Thirteen antimicrobial compounds belonging to various classes of antibiotics including β-lactam, quinolone, aminoglycoside, and polymyxin were used to generate the antibiogram profile. Within the β-lactam class, 92.3% of the isolates were resistant to amoxycilin and clavulanic acid followed by cefepime (84.61%) and ceftriaxone (53.8%) (Fig. 1). Among the aminoglycosides, 53.45% of the isolates showed resistance to gentamicin followed by netillin (34.6%). In the quinolone class, 46.15% and 34.61% isolates showed resistance to ciprofloxacin and olfloxacin respectively (Fig. 1). Colistin was found to be most effective because only 7.7% of the bacteria showed resistance to this antibiotic (Fig. 1).

**Figure 2:**
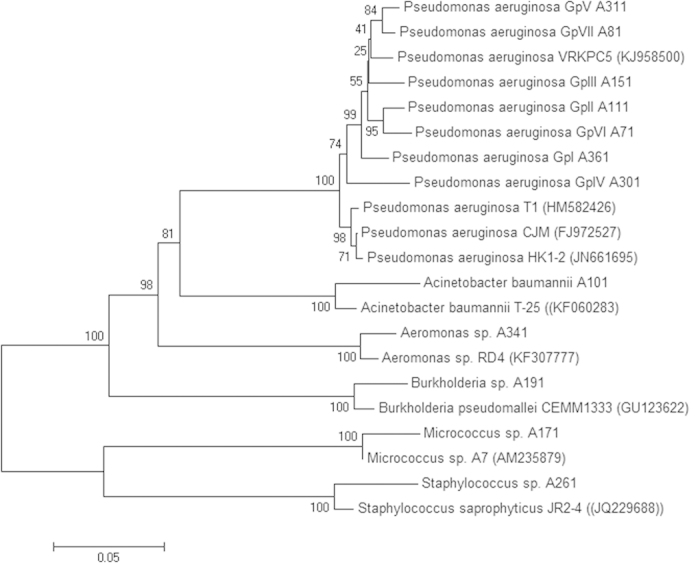
Evolutionary relationships of taxa. The evolutionary history was inferred using the Neighbor-Joining method. The percentage of replicate trees in which the associated taxa clustered together in the bootstrap test (1000 replicates) is shown next to the branches. The tree is drawn to scale, with branch lengths in the same units as those of the evolutionary distances used to infer the phylogenetic tree. The evolutionary distances were computed using the Maximum Composite Likelihood method and are in the units of the number of base substitutions per site. All ambiguous positions were removed for each sequence pair. Evolutionary analyses were conducted in MEGA6.

Identification of colistin resistant clinical isolates through 16S rRNA sequence analysis revealed predominance of *Pseudomonas aeruginosa* constituted (83.6%). Sequence similarity analysis clustered *Pseudomonas aeruginosa* into seven subgroups which were named as (GpI–GpVII) (Fig. 2). GpI showed 99% similarity to *Pseudomonas aeruginosa* NO2, GpII was 97% similar to *Pseudomonas aeruginosa* strain PA5-1-2, GpIII showed 98% similarity to *Pseudomonas aeruginosa* strain HK1-2, GpIV was 98% similar to *Pseudomonas aeruginosa* strain T1, GpV showed similarity to *Pseudomonas aeruginosa* strain VRKPC5, GpVI was 97% similar to *Pseudomonas aeruginosa* strain D2 and GpVII showed 98% similar to *Pseudomonas aeruginosa* strain NBAII AFP-7. Other groups identified were *Burkholderia pseudomallei* sp. A191 (5.17%), *Staphylococcus* sp. A261 (3.45%), *Micrococcus* sp. A171 (2.58%), *Aeromonas* sp. A341 (2.58%) and *Acinetobacter* sp. A341 (2.58%).

**Figure 3:**
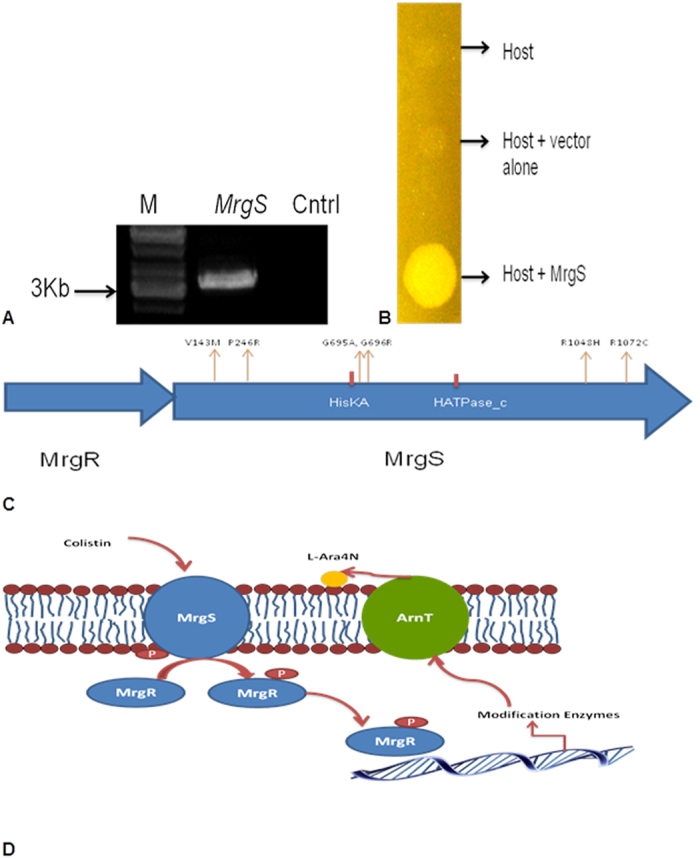
(**A**) PCR amplification of *MrgS* gene; (**B**) Growth profile of host, host with vector only and host with *MrgS* gene on colistin plate; (**C**) Domains of the MrgR/MrgS two component system and positions of all mutations in *MrgS* gene comferring colistin resistance. MrgS domains, “HisKA” - Histidine Kinase A dimerization/phosphoacceptor domain; “HATPase_c” - Histidine kinase-like ATPases. (**D**) Proposed model showing the activation of lipopolysaccharide-modifying genes involved in colistin resistance.

It was alarming to find that *Burkholderia pseudomallei* sp. A191 and *Pseudomonas aeruginosa* sp. A111 (GpII) showed resistance to higher concentration of colistin ie. 1500 μg/ml and 750 μg/ml respectively. MIC value of colistin for *B. pseudomallei* 1026b was reported as 128 mg/ml^16^. Cell free extracts of both the isolates however, did not show any enzymatic degradation of colistin (Table S1). Functional genomics library of *Burkholderia pseudomallei* sp. A191 and *Pseudomonas aeruginosa* sp. A111 were of size of 2.1 × 10^7^ bp and 1.30 × 10^6^ bp respectively. Five colistin-resistant clones were obtained after functional screening of *Burkholderia pseudomallei* sp. A191, but no resistant clone was observed in *Pseudomonas aeruginosa* sp. A111 library. Interestingly, after analysis of insert DNA sequences from all the five *Burkholderia pseudomallei* clones, it was observed that all clones have a common DNA sequence or gene that could be responsible for conferring colistin resistance. Based on ORF prediction, two components regulatory system encoding for a histidine kinase (mrgS) and its regulatory component (mrgR) were observed in the clone DNA sequence that showed 98–99% similarity with other histidine kinase sequences of *Burkholderia pseudomallei*. Histidine kianse (mrgs) was PCR amplified, sub-cloned and sequenced (Fig. 3A). Six point mutations were observed in *mrgS* gene viz. V143M, P246R, G695A, G696R, R1048H and R1072C (Fig. 3C). Resistance model based on two component regulatory system which controls the expression of genes responsible for LPS modification has been proposed (Fig. 3D). Colistin sensitive strain of *Burkholderia pseudomallei* was not available with us thus other Gram negative colistin sensitive species were explored for complementation assay. Transformation of *Pseudomonas aeruginosa* sp. A71 with mrgS plasmid causes the development of colistin resistant phenotype. Microdilution assay showed that *Pseudomonas aeruginosa* sp. A71+mrgS plasmid was able to grow with varying concentrations of colistin (≥100 μg/ml) as compared with *Pseudomonas aeruginosa* sp. A71 transformed with vector alone (Fig. 3B and Table 2).

## Discussion

The burden of infectious diseases in India is the highest in the world which gets further aggravated due to the inappropriate and irrational use of antimicrobial agents against these diseases, resulting in increased incidence of development of antimicrobial resistance. Surveillance of antimicrobial resistance in microbial populations associated with patients is not conducted in hospitals due to lack of proper infrastructure. Lack of information about the antimicrobial resistance patterns in patients presents an obstacle to disease management, with respect to antimicrobial therapy, patient prognosis, and infection control^9^,^10^. 16S rRNA sequencing of sample isolates to identify the diverse multidrug resistant bacteria such as *Burkholderia pseudomallei* sp. A191, *Micrococcus* sp. A7, *Aeromonas* sp. A341, *Staphylococcus* sp. A261 and different strains of *Pseudomonas aeruginosa* acted as an alternative to standard classical tools and to prevent the misidentification of clinically relevant isolates^11^,^12^.

All the bacteria identified in this study were found to be resistant to multiple classes of antibiotics though colistin was tested as the most effective drug against these bacteria. Among the isolates, *Burkholderia pseudomallei* sp. A191 and *Pseudomonas aeruginosa* sp. A111 showed high level colistin resistance (>500 ug/ml). *Burkholderia* species is known to be intrinsically resistant to colistin but whichever few reports of colistin resistance have been emerged out are centered on *Pseudomonas* aeruginosa and *Klebsiella pneumoniae*^13^. It is an alarming situation and clinicians should be aware that colistin resistance can occur in *P. aeruginosa*, and some of these strains have the capability for cross contamination with in a hospital set up. Occurrence of multi drug resistance in *P. aeruginosa* and capability of *P. aeruginosa* for cross contamination within a hospital set up is quite alarming to clinicians and also research scientist working in this field. Lee and Ko^14^ tried to correlate the two component regulatory systems PmrAB and PhoPQ with colistin resistance in *Pseudomonas aeruginosa.* However no amplification was obtained using the primers specific for TCRS based on *Pseudomonas aeruginosa* PA14 which could be attributed to difference with in the genomes at strain level (Data not shown). Alternatively, may be a different mechanism is prevailing in this bacterium responsible of resistance which is yet to be clearly identified. However, no reports on the mechanism of colistin resistance in *Burkholderia* sp. are available.

Various major and minor determinants of polymyxin B like; truncation of the LPS core oligosaccharide^15^, sigma factor RpoE^16^, zinc metalloproteases^17^ and an efflux system (NorM)^18^ have been proposed to be responsible for bringing resistance in *Burkholderia* sp. Among these determinants, two component response regulator (BCAL2831) and a periplasmic protease (MucD) are less studied in *Burkholderia cenocepacia*. Lack of potential of cell free extracts in catalytic degradation of colistin ruled out the role of protease in colistin conversion. External stimuli like pH or metal ions trigger the activation of TCRS^19^,^20^. Functional genome screening led us to discover the TCRS from *Burkholderia pseudomallei* sp. A191 and sequence analysis revealed unique mutations in *mrgS*. Since DNA sequences in all the clones were same, we hypothesized that mutations within the TCRS may result in the constitutive activation and subsequent expression of LPS modifying genes (Fig. 3D). This modification is carried out by the products of the *pmrHFIJKLM* operon, which is conserved among Enterobacteriaceae and is positively regulated by the PhoQ/PhoP and PmrAB signaling systems^21^,^22^.

To confirm this hypothesis, cross complementation assay were initiated which showed that mutations in two component regulatory system mrgRS of *Burkholderia pseudomallei* sp. A191 were sufficient to confer colistin resistance in the sensitive strain of *Pseudomonas aeruginosa* sp. A71. Non availability of colistin sensitive *Burkholderia* sp. forced us to do complementation assay with *Pseudomonas aeruginosa* sp. A71. To date it has not been possible to isolate the *Burkholderia* sp. which showed sensitivity to polymyxins^16^. Cross complementation assay demonstrated the possibility of dissemination of colistin resistant determinant among the clinical isolates within a hospital.

It can be concluded that regular surveillance to track the clinical isolates with correct identification is highly important to tackle the antibiotic resistant pathogens. Discovery of colistin resistance at such a high concentration is alarming which highlights the need for strict measures to keep a tab on judicious antibiotic usage for infection control. Genetics of colistin resistance showed that single gene mutations are sufficient to confer the resistance. Cross complementation assays demonstrated that spread of resistant determinants is highly possible within a hospital.

## Methodology

### Sample collections

Institute of Medical Sciences and Sum Hospital (IMS & SUM), Siksha “O” Anusandhan University, Bhubaneswar, Odisha, is a more than 1000 bed hospital and handles 1500 patients on daily basis. Samples were collected from hospitalized-patients admitted to different wards of hospital during August 2012 to September 2013 (Table 1). Primary identification of bacteria was done based on the standard conventional morphological and biochemical tests.

### Ethical Considerations

All participants were recruited under informed consent form in accordance with the approved guidelines from “Indian Council of Medical Research”, signed either by the patient or their family member. All experimental protocols were approved by the Institutional Ethical Committee, Center of Biotechnology (School of Pharmaceutical Sciences), SOA University, India. All the experimental methods were carried out in accordance with the approved guidelines.

### Antimicrobial susceptibility testing

The susceptibility of these isolates was tested against antimicrobial agents according to the Clinical and Laboratory Standards Institute (CLSI) guidelines (2015). End-points were read after overnight incubation at 37 °C. The test microbes were taken from the broth culture with inoculating loop and transferred to test tubes containing 5.0 ml sterile distilled water. The inoculums were added until the turbidity became equal to 0.5 McFarland standards. Cotton swab was then used to inoculate the test tube suspension onto the surface of the Muller Hinton agar plate and the uniformly swabbed plates were then allowed to dry. Antimicrobial agents and ranges (μg/ml) tested were: amoxycillin/clavulanic acid (4–32), ciprofloxacin(0.125–8), ceftriaxone (8–64), amikacin (1–64), ceftazidime (16–32), cefepime (2–32), gentamicin (2–16), netin (4–32), cefoperazone (0.05–64), tobramycin (2–16), ofloxacin (2–8), imipenem (0.5–16), Cefpirome (0.25–4) and colistin (0.125–32). *P. aeruginosa* ATCC 27853 was used as a control strain.

### Molecular identification of antibiotic resistant bacteria

Chromosomal DNA of the bacteria was extracted as described by Kumar *et al.*^23^. 16S rRNA gene was PCR amplified using genomic DNA as template with universal primers E8F (AGAGTTTGATCCTGGCTCAG) and reverse primer E1492R (GGT-TACCTTGTTACGACTT)^24^. The PCR reactions were prepared as described by Kumar *et al.*^25^ and thermal cycling was performed as described by Kumar *et al.*^23^. Sequencing was done by Amnion Biosciences, Bangalore, India and then BLAST searched through the NCBI GenBank database. Phylogenetic tree was constructed using molecular evolutionary genetics analysis (MEGA) software with 1000 bootstrap replicates^26^.

### Enzymatic assay

Cell extracts were incubated with colistin (100μg/ml) in a buffer. After regular interval of time, samples were analyzed by HPLC for the estimation of degradation of colistin. HPLC Prominence system (Shimadzu, Singapore) equipped with a binary LC-20AD pumping system with an online vacuum degasser, SIL-20 autosampler, and SPD-M20A photodiode array detector (PDA) detector was applied to chromatographic studies. Chromatographic separations of colistin were achieved on the C18 column (150mm × 4.6 mm i.d.). Phenomenex, USA). Samples were filtered through a Millipore membrane (0.45 μm). LCsolution software was used for data acquisition and integration. The UV detector was set at 214 nm and the temperature was ambient temperature. The sample injection volume of the autosampler was 5.0 μL. The chromatographic separation was performed using a linear gradient (20% B (acetonitrile)+80% A (0.05% TFA aqueous solution) changed to 50% B+50% A in 10 min).

### Genomic expression library construction

A genomic expression library was constructed by shearing the genomic DNA (gDNA) in order to obtain 7.0–9.0 kb DNA fragments after agarose gel electrophoresis. The sheared DNA fragments were end-repaired and ligated into a pUC-19 plasmid and were then electrotransformed into colistin sensitive *Pseudomonas aeruginosa* sp. A71 as host. Insert size distribution was estimated by gel electrophoresis of colony PCR products obtained by amplifying the insert using vector specific primers. The total size of the genomic expression library was determined by multiplying average PCR based insert size by the number of total CFUs obtained. The transformation mixture was enriched by growing the cells in selective LB broth and glycerol preserved at −70 °C until further processing. Resistant clones containing unique DNA inserts were sequenced using Sanger sequencing technology (Amnion Biosciences, Bangalore, India).

### Molecular cloning of two component regulatory systems

Recombinant plasmid was extracted from a resistant clone. Oligonucleotides were designed to amplify the two component regulatory system (TCRS) *mrgRS* coding for transcriptional regulator and kinase sensor using plasmid as template.

B.mrgR-F 5′ATGTCCAACGTTGCCCTGCATAC3′

B.mrgR-R 5′CAGAGCAGTCCTTGCTCGCGGG3′

B.mrgS-F 5′ATGCAAGGACTTCTGCAAGAGCT3′

B.mrgS-R 5′TCAGCGCCGCGACCATTCGCTCC3′

PCR amplification was performed as described above. Amplicon was ligated into pTZ57R vector (pUC based) as per manufacturer’s instructions and transformed into electrocompetent *Pseudomonas aeruginosa sp.* A71^27^. The presence of this plasmid in the colonies was confirmed using PCR with universal M13 primers where the amplification of the product in this PCR required the junction between the *mrgS* sequence and the vector to be present. The sequence homology search and conserved domain analysis of the deduced protein sequence were performed using the BLASTP program (http://blast.ncbi.nlm.nih.gov/Blast.cgi) and CDART program (http://www.ncbi.nlm.nih.gov/Structure/lexington/lexington.cgi) of NCBI, respectively. The ORFs were identified by using the NCBI’s open reading frame (ORF) Finder tool (http://www.ncbi.nlm.nih.gov/gorf/gorf.html).

### Cross complementation assay

To determine whether the mutations in *mrgS* were sufficient to confer colistin resistance, a broth microdilution assay was used to determine the MIC of sensitive strain *Pseudomonas aeruginosa* A71 carrying the empty vector or the vector with the *mrgS* gene. Overnight culture of *Pseudomonas aeruginosa* A71 grown in Mueller-Hinton broth (MHB) plus carbenicillin (200 μg/ml) was harvested by centrifugation, washed with phosphate buffer, re-suspended in MHB broth, and inoculated into MHB with a series of colistin concentrations.

### Nucleotide sequence submission

The nucleotide sequences of 16S rDNA of the clinical bacterial strains reported in this study was deposited in the GenBank database with the following accession numbers *Acinetobacter baumanii* (KT819271), *Micrococcus* sp. A171 (KT819272), *Burkholderia pseudomallei* A191 (KT819273), *Staphylococcus* sp. A261 (KT819274), *Aeromonas* sp. A341 (KT819275), *Pseudomonas aeruginosa* A361 (KT819276), *Pseudomonas aeruginosa* A111 (KT819277), *Pseudomonas aeruginosa* A151 (KT819278), *Pseudomonas aeruginosa* A301 (KT819279), *Pseudomonas aeruginosa* A311 (KT819280), *Pseudomonas aeruginosa* A71 (KT819281), *Pseudomonas aeruginosa* A81 (KT819282).

## Additional Information

**How to cite this article**: Kumar, M. *et al.* Functional Genome Screening to Elucidate the Colistin Resistance Mechanism. *Sci. Rep.* **6**, 23156; doi: 10.1038/srep23156 (2016).

## Supplementary Material

Supplementary Information

## Figures and Tables

**Table 1:** Information of the patients and source of sampling of clinical bacterial isolates.

Isolates	Sex	Age	Source
A51	F	24	wound
A71	M	22	wound
A81	M	18	wound-pus
A101	M	70	wound
A111	M	52	sputum
A121	M	60	head injury
A141	M	52	non-healing Ulcers
A151	F	75	pus
A171	M	44	wound
A181	M	06	urine
A191	F	20	pus
A221	M	22	pus
A251	F	20	60%burn
A261	F	20	wound
A271	F	20	wound
A281	M	58	pus
A291	M	26	urine
A301	F	50	pus
A311	M	40	pus
A321	F	27	urine
A331	F	14	pus
A341	M	40	pus
A351	M	50	pus
A361	F	66	urine
A371	F	57	fish eye
A381	M	70	traumatic burning injury

**Table 2:** Cross complementation of *mrgS* mutations.

Strain	Colistin resitance(μg/ml)
*Pseudomonas aeruginosa* A71+vector	<0.5
*Burkholderia pseudomallei* A91	≥100
*Pseudomonas aeruginosa* A71+mrgRS	≥100

## References

[b1] Alekshun M. N. & Levy S. B. Molecular mechanisms of antibacterial multidrug resistance. Cell 128, 1037–50 (2007). 17382878 10.1016/j.cell.2007.03.004

[b2] Gold H. S. & Moellering R. C. Antimicrobial-drug resistance. N. Engl. J. Med. 335, 1445–53 (1996). 8875923 10.1056/NEJM199611073351907

[b3] Peacock S. Health care: Bring microbial sequencing to hospitals. Nature 509, 557–559 (2014). 24877182 10.1038/509557a

[b4] Moffatt J. H. *et al.* Colistin resistance in Acinetobacter baumannii is mediated by complete loss of lipopolysaccharide production. Antimicrob. Agents Chemother. 54, 4971–7 (2010). 20855724 10.1128/AAC.00834-10 PMC2981238

[b5] Ah Y.-M., Kim A.-J. & Lee J.-Y. Colistin resistance in Klebsiella pneumoniae. Int. J. Antimicrob. Agents 44, 8–15 (2014). 24794735 10.1016/j.ijantimicag.2014.02.016

[b6] Wright M. S. *et al.* Genomic and transcriptomic analyses of colistin-resistant clinical isolates of Klebsiella pneumoniae reveal multiple pathways of resistance. Antimicrob. Agents Chemother. 59, 536–43 (2015). 25385117 10.1128/AAC.04037-14 PMC4291396

[b7] Cheng Y.-H. *et al.* Colistin resistance mechanisms in Klebsiella pneumoniae strains from Taiwan. Antimicrob. Agents Chemother. 59, 2909–13 (2015). 25691646 10.1128/AAC.04763-14 PMC4394772

[b8] Taneja N., Singh G., Singh M. & Sharma M. Emergence of tigecycline & colistin resistant Acinetobacter baumanii in patients with complicated urinary tract infections in north India. Indian J. Med. Res. 133, 681–4 (2011). 21727671 PMC3136000

[b9] Pallen M. J., Loman N. J. & Penn C. W. High-throughput sequencing and clinical microbiology: progress, opportunities and challenges. Curr. Opin. Microbiol. 13, 625–31 (2010). 20843733 10.1016/j.mib.2010.08.003

[b10] Long S. W. *et al.* A genomic day in the life of a clinical microbiology laboratory. J. Clin. Microbiol. 51, 1272–7 (2013). 23345298 10.1128/JCM.03237-12 PMC3666789

[b11] Boudewijns M., Bakkers J. M., Sturm P. D. J. & Melchers W. J. G. 16S rRNA gene sequencing and the routine clinical microbiology laboratory: a perfect marriage? J. Clin. Microbiol. 44, 3469–70 (2006). 16954306 10.1128/JCM.01017-06 PMC1594676

[b12] Rhoads D. D., Cox S. B., Rees E. J., Sun Y. & Wolcott R. D. Clinical identification of bacteria in human chronic wound infections: culturing vs. 16S ribosomal DNA sequencing. BMC Infect. Dis. 12, 321 (2012). 23176603 10.1186/1471-2334-12-321 PMC3542000

[b13] Johansen H. K., Moskowitz S. M., Ciofu O., Pressler T. & Høiby N. Spread of colistin resistant non-mucoid Pseudomonas aeruginosa among chronically infected Danish cystic fibrosis patients. J. Cyst. Fibros. 7, 391–397 (2008). 18358794 10.1016/j.jcf.2008.02.003

[b14] Lee J.-Y. & Ko K. S. Mutations and expression of PmrAB and PhoPQ related with colistin resistance in Pseudomonas aeruginosa clinical isolates. Diagn. Microbiol. Infect. Dis. 78, 271–6 (2014). 24412662 10.1016/j.diagmicrobio.2013.11.027

[b15] Ortega X. P. *et al.* A Putative Gene Cluster for Aminoarabinose Biosynthesis Is Essential for Burkholderia cenocepacia Viability. J. Bacteriol. 189, 3639–3644 (2007). 17337576 10.1128/JB.00153-07 PMC1855895

[b16] Loutet S. A. & Valvano M. A. Extreme antimicrobial Peptide and polymyxin B resistance in the genus burkholderia. Front. Microbiol. 2, 159 (2011). 21811491 10.3389/fmicb.2011.00159 PMC3143681

[b17] Kooi C. & Sokol P. A. Burkholderia cenocepacia zinc metalloproteases influence resistance to antimicrobial peptides. Microbiology 155, 2818–25 (2009). 19542010 10.1099/mic.0.028969-0

[b18] Kuroda T. & Tsuchiya T. Multidrug efflux transporters in the MATE family. Biochim. Biophys. Acta 1794, 763–8 (2009). 19100867 10.1016/j.bbapap.2008.11.012

[b19] Abraham N. & Kwon D. H. A single amino acid substitution in PmrB is associated with polymyxin B resistance in clinical isolate of Pseudomonas aeruginosa. FEMS Microbiol. Lett. 298, 249–54 (2009). 19663916 10.1111/j.1574-6968.2009.01720.x

[b20] Miller A. K. *et al.* PhoQ mutations promote lipid A modification and polymyxin resistance of Pseudomonas aeruginosa found in colistin-treated cystic fibrosis patients. Antimicrob. Agents Chemother. 55, 5761–9 (2011). 21968359 10.1128/AAC.05391-11 PMC3232818

[b21] Cheng H.-Y., Chen Y.-F. & Peng H.-L. Molecular characterization of the PhoPQ-PmrD-PmrAB mediated pathway regulating polymyxin B resistance in Klebsiella pneumoniae CG43. J. Biomed. Sci. 17, 60 (2010). 20653976 10.1186/1423-0127-17-60 PMC2919465

[b22] Cannatelli A. *et al.* *In vivo* emergence of colistin resistance in Klebsiella pneumoniae producing KPC-type carbapenemases mediated by insertional inactivation of the PhoQ/PhoP mgrB regulator. Antimicrob. Agents Chemother. 57, 5521–6 (2013). 23979739 10.1128/AAC.01480-13 PMC3811314

[b23] Kumar M., Joshi A., Kashyap R. & Khanna S. Production of xylanase by Promicromonospora sp MARS with rice straw under non sterile conditions. Process Biochem. 46, 1614–1618 (2011).

[b24] Bond P., Hugenholtz P., Keller J. & Blackall L. Bacterial community structures of phosphate-removing and non-phosphate- removing activated sludges from sequencing batch reactors. Appl. Envir. Microbiol. 61, 1910–1916 (1995). 10.1128/aem.61.5.1910-1916.1995 PMC167453 7544094

[b25] Kumar M., Revathi K. & Khanna S. Biodegradation of cellulosic and lignocellulosic waste by Pseudoxanthomonas sp R-28. Carbohydr. Polym. 134, 761–766 (2015). 26428183 10.1016/j.carbpol.2015.08.072

[b26] Tamura K., Stecher G., Peterson D., Filipski A. & Kumar S. MEGA6: Molecular Evolutionary Genetics Analysis version 6.0. Mol. Biol. Evol. 30, 2725–9 (2013). 24132122 10.1093/molbev/mst197 PMC3840312

[b27] Kumar M. & Khanna S. Shift in microbial population in response to crystalline cellulose degradation during enrichment with a semi-desert soil. Int. Biodeterior. Biodegradation 88, 134–141 (2014).

