# Supplementary material for: Functional Genome Screening to Elucidate the Colistin Resistance Mechanism
Source: Sci Rep. 2016 Mar 18;6:23156. doi: 10.1038/srep23156 (PMC4796810; doi:10.1038/srep23156)
Supplement: Supplementary Information [file srep23156-s1.doc]

**Functional Genome Screening to Elucidate the Colistin Resistance Mechanism**

Mohit Kumar^1^*, Ashutosh Gupta^1#^, Rajesh Kumar Sahoo^3#^, Jayanti Jena^2#^, Nagen Kumar Debata^2^, Enketeswara Subudhi^3^

^1^Biotechnology and Bioinformatics, NIIT University, Neemrana, Rajasthan 301705, India

^2^Department of Microbiology, IMS & SUM Hospital, Siksha ‘O’ Anusandhan University, Kalinga Nagar, Bhubaneswar, Odisha 751003, India

^3^Centre of Biotechnology, Siksha ‘O’ Anusandhan University, Khandagiri, Bhubaneswar, Odisha 751003, India

Table S1: Enzymatic assay for colistin analyzed by HPLC.

| Time (mins) | Total Peak Area (Control) | Total Peak Area (Enzyme assay) | Degradation (%) |
| --- | --- | --- | --- |
| 0 | 2890 | 2889 | Nil |
| 30 | 2901 | 2902 | Nil |
| 60 | 2893 | 2891 | Nil |
| 120 | 2891 | 2893 | Nil |
